# Imputation of urban environmental sensor data using gated attention bidirectional long short-term memory (GA-BiLSTM): methods, performance, and implications

**DOI:** 10.1007/s10661-026-15112-8

**Published:** 2026-02-27

**Authors:** Jangho Lee, Max Berkelhammer, Joseph O’Brien, Gavin McNicol, Anna E. S. Vincent, Maxwell Grover, Aaron I. Packman, Bilal Kaludi, Ahram Cho, Miquel Gonzalez-Meler

**Affiliations:** 1 Department of Earth and Environmental Sciences, University of Illinois Chicago, Chicago, IL 60607 USA; 2 Environmental Research Division, Argonne National Laboratory, Lemont, IL 60439 USA; 3 Department of Civil & Environmental Engineering, Northwestern University, Evanston, IL 60208 USA; 4 Northwestern Center for Water Research, Northwestern University, Evanston, IL 60208 USA; 5 Department of Biological Sciences, University of Illinois Chicago, Chicago, IL 60607 USA

**Keywords:** Environmental monitoring, Sensor networks, Data imputation, Data quality, GA-BiLSTM

## Abstract

Urban environmental monitoring networks frequently encounter significant data gaps due to sensor malfunctions, environmental disturbances, and communication failures. Reliable approaches to address these gaps are essential for ensuring the continuity and quality of environmental data streams. In this study, we developed a gated attention bidirectional long short-term memory (GA-BiLSTM) model to impute missing data in a dense urban monitoring network. Using observations from the CROCUS network in Chicago, we evaluated GA-BiLSTM against widely used approaches (XGBoost and K-nearest neighbors) under scenarios of both short-term intermittent gaps and prolonged outages. GA-BiLSTM consistently outperformed comparative methods, particularly during extended outages of up to ten days, demonstrating its ability to capture spatiotemporal dependencies across sensor nodes. Beyond performance metrics, feature importance and spatial network analyses highlighted the unexpected but critical predictive role of peripheral rural nodes, underlining their strategic value for maintaining robust urban monitoring systems. These results emphasize that advanced imputation methods can substantially improve the reliability of environmental monitoring networks and support more resilient data infrastructures for urban sustainability.

## Introduction

Urbanization has accelerated dramatically in recent decades, with more than 70% of the world’s population now living in cities. This rapid growth has created complex urban microclimates—fine-scale variations in air temperature, humidity, wind patterns, and air quality, shaped by dense built environments and human activities (Huang & Song, 2023; Lee, 2024; Lee & Berkelhammer, 2024; Yang et al., 2023; Zhang et al., 2022; Zhao et al., 2023). Monitoring these microclimates is essential for understanding urban phenomena such as urban heat islands, pollution hotspots, and localized extreme weather. Additionally, subsurface environmental conditions, including soil temperature or volumetric water content (VWC), play critical roles in urban ecosystems, impacting urban vegetation health, stormwater management, groundwater recharge, and the overall resilience of urban infrastructure to climatic stressors (Anni et al., 2020; Brandani et al., 2016; Qin, 2020; Umer et al., 2019; Wu et al., 2014). High-resolution environmental data that include both atmospheric and soil variables can directly inform climate adaptation strategies, public health interventions, and urban green infrastructure planning (Creutzig et al., 2019; Zölch et al., 2016). However, capturing the true dynamics of a city’s environmental conditions—both at the surface and below ground—requires dense networks of ground-level sensors that resolve micro-scale variability at the neighborhood or even street scale (Brown et al., 2018; Chen & Yang, 2022; Šećerov et al., 2019).

Distributed, ground-based sensor networks represent a critical tool for observing urban environments with high temporal and spatial resolution (Jha et al., 2015; Muller et al., 2013). In contrast to satellite remote sensing or coarse-resolution reanalysis products, in-situ sensor nodes provide direct measurements of both near-surface atmospheric variables and subsurface conditions, at precise locations, and with fine temporal granularity. While satellite observations offer broader spatial coverage, they often lack the necessary resolution to distinguish micro-scale urban features and can only indirectly infer subsurface conditions. Similarly, reanalysis products deliver continuous but relatively coarse-scale coverage, typically at kilometer resolutions, insufficient to capture detailed urban variability—especially subsurface variations that critically influence urban environmental dynamics.

Despite their critical benefits, urban sensor networks frequently encounter a significant challenge: missing data. Real-world deployments often experience gaps in data streams due to sensor outages, power failures, human interference, maintenance activities, and communication disruptions (Kong et al., 2013). Dense urban environments introduce additional challenges, including signal interference, harsh environmental conditions, such as urban flooding, which disproportionately affect ground-level and subsurface sensors, and vandalism. Consequently, it is common for urban sensor nodes to experience intermittent data losses or extended downtime. These data gaps interrupt continuous environmental monitoring, limiting effective data analysis, and potentially biasing subsequent scientific conclusions.

A variety of imputation techniques have been developed to fill these gaps in environmental datasets. Simple methods, such as mean substitution, forward filling, or linear interpolation, can handle short-term outages but often distort data variability and underestimate extremes (Decorte et al., 2024). Advanced statistical and geostatistical approaches (e.g., ARIMA models, Kalman filters, kriging, or inverse-distance weighting) exploit temporal and spatial correlations to improve imputation (Afrifa‐Yamoah et al., 2020; Bianchi et al., 1999; Kyrtsoglou et al., 2021; Moritz & Bartz-Beielstein, 2017; Walter et al., 2013). Yet, these approaches typically assume relatively short and infrequent gaps and rely heavily on linear assumptions and/or stationary relationships, resulting in reduced accuracy for prolonged outages or strongly nonlinear environmental patterns, as frequently observed in urban soil moisture and temperature data.

Machine learning approaches offer enhanced flexibility, capable of modeling complex nonlinear relationships in the data. Methods such as K-nearest neighbors (KNN) and random forests have been applied to environmental datasets to predict missing values based on available spatial and temporal features (Almeida et al., 2024; Arnaut et al., 2024; Belachsen & Broday, 2022; Kiani & Saleem, 2017; Mital et al., 2020). Matrix factorization and tensor completion methods treat sensor network data as partially observed matrices or tensors, extracting latent factors representing spatial and temporal patterns.

More recently, deep learning models—particularly long short-term memory (LSTM) based networks—have demonstrated strong capabilities in modeling complex sequential relationships in sensor data, including soil conditions (Rani & Solanki, 2021; Tzoumpas et al., 2024; Wang et al., 2025; Yu et al., 2021). Some particular derivatives of LSTM architecture include Bidirectional recurrent imputation for time series (BRITS), often used for time series imputation (Cao et al., 2018; Decorte et al., 2024). Also, autoencoder based (Ba-Alawi et al., 2022; Pereira et al., 2020) or generative adversarial network (GAN) based methods (Li et al., 2025; Zhong et al., 2024) have been gaining attention with great promise.

However, one domain-related aspect in urban soil sensors is that sensors distributed within an urban area do not measure environmental conditions in isolation. Variables like soil temperature, VWC, and vapor pressure deficit (VPD) exhibit strong spatial correlations, as nearby sensors often reflect similar trends and respond collectively to meteorological events such as precipitation, heatwaves, or storms. These interrelationships provide an opportunity to improve imputation: if a particular soil sensor experiences an outage, measurements from nearby sensors measuring either the same property or a correlated property can provide valuable contextual information to more accurately infer the missing data. Effective imputation therefore requires dynamically combining inputs from multiple neighboring sensors, balancing their relative importance, and suppressing noisy or less informative measurements.

In this work, we introduce a gated attention bidirectional long short-term memory (GA-BiLSTM) model specifically designed to address missing data in urban environmental sensor networks. Our model explicitly leverages temporal dependencies within individual sensors and spatial relationships across multiple nodes. The GA-BiLSTM processes sensor measurements bidirectionally, enabling the network to incorporate contextual information from observations both preceding and following data gaps. This combined spatiotemporal approach ensures context-aware imputations, accurately capturing local soil dynamics and broader patterns in urban environmental conditions. Beyond methodological advancements, we perform further analyses to derive scientific insights into the underlying spatial–temporal relationships among sensor nodes. These insights contribute valuable knowledge to inform strategic sensor deployment decisions, enhancing both the efficiency and effectiveness of urban environmental monitoring networks.

## Data

### CROCUS sensor measurements

**Fig. 1:**
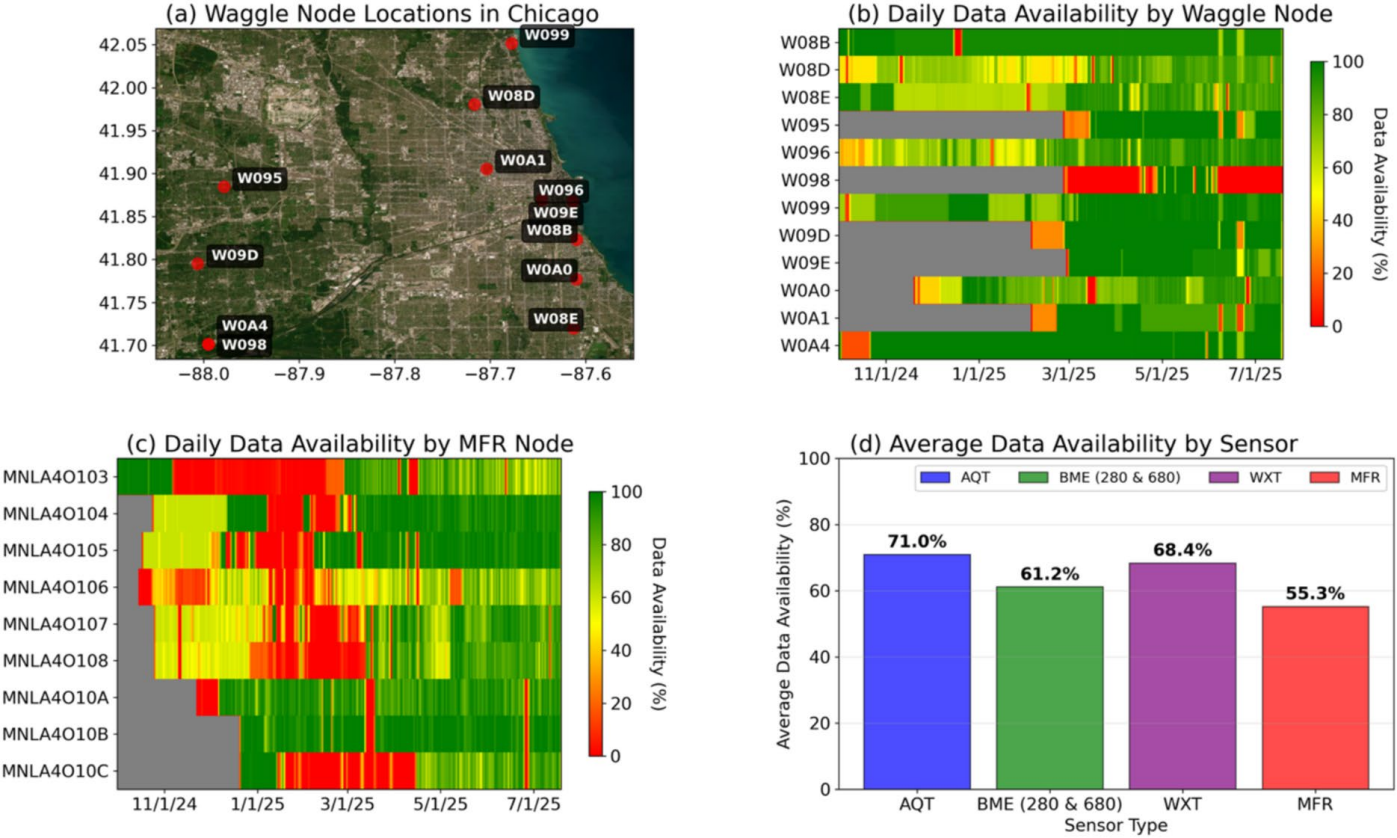
**a** Locations and IDs of the 12 Waggle node sites in the Chicago metropolitan area. **b** Operational status (%) by site, indicating data availability. Grey area represents the pre-installation period for each node. **c** Operational status (%) specifically for MFR nodes. **d** Average data availability by sensor type

CROCUS is one of the U.S. Department of Energy’s Urban Integrated Field Laboratory (UIFL) projects and it was designed for comprehensive environmental monitoring and modeling within the Chicago metropolitan area. Through this project, a network of atmospheric and in-ground sensors has been strategically deployed around Chicago region to capture diverse urban conditions (O’Brien et al., 2024a, b, c). Although the majority of sensors were installed after October 2024, some deployments extend as far back as May 2022, enabling both historical data comparisons and the analysis of long-term environmental patterns. In this study, we specifically focus on data from 12 selected sites that have comprehensive data on above and belowground conditions (Fig. 1a).

The cyberinfrastructure backbone of the sensor network is provided by an advanced integrated sensor platform called a “Waggle node,” installed at each of these sites, predominantly on rooftops, towers, or tripods. Waggle nodes are outfitted with multiple sensors to measure key meteorological and air-quality variables, including barometric pressure, humidity, air temperature, wind direction and speed, precipitation, and atmospheric pollutants (Table 1). Waggle nodes communicate with the main server via hard line ethernet or wifi, where about 1/3 of the nodes are using cellular modems. Most of the nodes include a Long Range Wide Area Network (LoRaWAN) gateway (de Carvalho Silva et al., 2017; Haxhibeqiri et al., 2018) and antennas that enable them to assimilate data via telemetry from distributed LoRaWAN-enabled sensors within ~ 2 km that are connected to data loggers (so-called MFR nodes, as described below).

**Table 1:** Summary of sensor types, measurement frequency, and the environmental variables each sensor measure at Waggle and MFR node

Sensor	Measurement frequency	Measuring variable
BME280	0.5 min	Barometric pressure, relative humidity, air temperature (core temperature)
BME680	0.5 min	Barometric pressure, relative humidity, air temperature (shield temperature)
AQT530	0.3 min	Concentration of atmospheric pollutant & particulate matter (CO, NO, NO2, O_3_, PM1, PM2.5, PM10), barometric pressure, humidity, air temperature
WXT536	0.25 s	Barometric pressure, humidity, air temperature, rain accumulation, wind direction, wind speed
MFR Nodes	15 min	Air temperature, ground heat flux, shortwave radiation (in, out, and net), longwave radiation (in, out, and net), soil temperature (at 15 cm, 30 cm, 45 cm, and 60 cm depth), soil volumetric water content (VWC, at 15 cm, 30 cm, 45 cm, and 60 cm depth), vapor pressure deficit (VPD), water conductivity, water depth, water temperature

Complementing these rooftop Waggle nodes are Multi-Function Remote (MFR) nodes that are strategically placed at ground level across five of the 12 sites (Table 2). These nodes provide observations of surface and subsurface environmental conditions, capturing detailed soil and hydrological data. The deployment of MFR nodes includes multiple soil sensors capable of measuring soil temperature and volumetric water content (VWC) at varying depths (15, 30, 45, and 60 cm), vapor pressure deficit (VPD), soil heat flux, and other relevant soil parameters (Teros54 and HFP01). Associated hydrological sensors (Hydros21) record groundwater depth, specific conductivity, and temperature.

**Table 2:** List of Waggle node sites and location with installed sensor types and number of associated MFR nodes (MNL identifiers, if applicable)

Waggle node	Lat	Lon	Sensors and MFR nodes
W0A4	41.7014	−87.9952	BME280, BME680, AQT530, WXT536
W0A1	41.9055	−87.7033	BME280, BME680, AQT530, WXT536
W0A0	41.7770	−87.6097	BME280, BME680, AQT530, WXT536, MNLA4O10A, MNLA4O10B, MNLA4O10C
W09E	41.8681	−87.6133	BME280, BME680, AQT530, WXT536
W09D	41.7952	−88.0061	BME280, BME680, AQT530, WXT536
W099	42.0514	−87.6776	BME280, BME680, AQT530, WXT536, MNLA4O104
W098	41.7013	−87.9948	BME280, BME680
W096	41.8694	−87.6458	BME280, BME680, AQT530, WXT536, MNLA4O105, MNLA4O106
W095	41.8848	−87.9787	BME280, BME680
W08E	41.7198	−87.6128	BME280, BME680, AQT530, WXT536, MNLA4O102, MNLA4O103
W08D	41.9805	−87.7166	BME280, BME680, AQT530, WXT536, MNLA4O107, MNLA4O108
W08B	41.8229	−87.6096	BME280, BME680, AQT530, WXT536

However, MFR nodes do not have wired power (using solar panels) and only transmit data via LoRaWAN so they experience higher vulnerability to operational disruptions, data loss, and sensor downtime compared to Waggle nodes. This vulnerability primarily comes from exposure to dynamic environmental conditions, physical disturbances, signal obstructions, and urban infrastructure interference. For example, during extended cold periods with limited sunlight, the batteries struggle to hold charges and the MFR nodes shut down. Furthermore, MFR nodes rely on longer-range communication with rooftop-based LoRaWAN transmitters, which contributes to potential instability in data transmission. In other words, they go down if either the MFR or Waggle node goes down.

Figure 1b quantitatively illustrates operational uptime and downtime for each site (Waggle and MFR combined), while Fig. 1c shows the operational status with emphasis on the variability observed among MFR nodes. Additionally, Fig. 1d provides further statistics into sensor reliability by showing the average availability of valid data across sensor types. As can be seen in the figure, the MFR nodes consistently exhibit higher rates of downtime compared to sensors installed in the Waggle node.

### Specifications of Waggle and MFR node sensors

In this section, we detail the specifications for sensors included in Waggle and MFR nodes. The variables measured by each sensor are listed in Table 1, and the specific sensors deployed at each site are summarized in Table 2. Waggle nodes may include all or part of the following sensors: Bosch BME280, Bosch BME680, Vaisala AQT530, and Vaisala WXT536.

The Bosch BME280 is a compact, low-power digital sensor capable of measuring relative humidity, atmospheric pressure, and temperature. It supports both SPI and I^2^C communication protocols, offers configurable oversampling and filtering options for improved accuracy, and is ideal for weather monitoring, indoor navigation, and IoT applications. Similarly, the Bosch BME680 is a compact, low-power 4-in-1 digital sensor designed for measuring gas (air quality), pressure, temperature, and humidity. It utilizes electrochemical gas sensor technology and a laser particle counter (LPC), providing real-time air quality monitoring. The Vaisala AQT530 is an air quality transmitter that measures pollutant gases (NO₂, NO, O₃, CO) and particulate matter (PM10, PM2.5, PM1) using advanced electrochemical gas sensors and laser particle counting technology. It incorporates intelligent humidity management, providing reliable performance suitable for urban air quality networks and roadside monitoring. Lastly, the Vaisala WXT536 is a versatile weather transmitter that provides measurements of key meteorological parameters: barometric pressure, air temperature, relative humidity, rainfall, wind speed, and wind direction, using ultrasonic sensors for wind, Vaisala HUMICAP technology for humidity, and Vaisala RAINCAP acoustic sensors for precipitation, ensuring high accuracy and maintenance-free operations suitable for diverse weather monitoring applications (Kyrouac & Tuftedal, 2024).

MFR nodes incorporate sensors specialized for measuring soil, thermal, and hydrological conditions, specifically the Teros54 (METER Group, Pullman, WA, USA), HFP01 (ICT International, Armidale, NSW, AUS), and Hydros21 (METER Group, Pullman, WA, USA) sensors, respectively. The METER Teros54 is a robust sensor that accurately measures soil moisture and temperature at four points along a depth profile, providing reliable data critical for monitoring soil conditions under varied environmental contexts. The Hydros21, also from METER, measures groundwater depth, temperature, and electrical conductivity. The Hukseflux HFP01 heat flux sensor is employed to measure soil heat flux, facilitating precise assessment of soil thermal properties and energy balance essential for understanding heat exchanges at the soil surface. These sensors collectively enable comprehensive monitoring of environmental and soil-related parameters, enhancing data quality and coverage for detailed analyses of site-specific conditions.

### Objective and data preprocessing

The primary objective of this study is to effectively impute missing data from the MFR nodes, which provide critical near-surface and subsurface environmental measurements. Especially, our objective is to impute the following variables from the MFR nodes: air temperature (AT), vapor pressure deficit (VPD), soil temperature at 15, 30, 45, and 60 cm depth (ST15, ST30, ST45, ST60), and volumetric water content at 15, 30, 45, and 60 cm depth (VWC15, VWC30, VWC45, VWC60). To accomplish this, we utilize complementary spatial and temporal information from the broader urban environmental sensor network. Spatial context is derived from observations at distributed sensor nodes strategically placed across the monitoring network, whereas temporal context leverages sensor measurements immediately preceding and following data gaps.

Prior to data imputation, quality control (QC) was implemented using a suite of physics-based methods. These methods include physical range checks to identify sensor malfunctions or extreme environmental conditions; step spike detection targeting sudden power fluctuations, electronic interference, or transient communication errors; 24-h flat-line checks to detect complete sensor failures or blockages; monotone ramp identification for sensor drift or calibration issues; jitter detection for sensor instability or electronic noise; ultra-low variance checks indicating sensor degradation, physical obstructions, or prolonged communication failures; high-frequency flip detection for unstable sensor responses; and persistent high-offset identification targeting sensor calibration drift or physical shielding damage. These QC measures collectively removed approximately 3% of the original data, eliminating clearly erroneous or physically implausible readings and thereby enhancing the reliability of subsequent imputation analyses. Following QC, all sensor measurements were synchronized to a standardized 30-min interval to address the inherent differences in data collection frequencies among sensor types (Table 1).

Given the primary focus of this study on imputation rather than forecasting, we developed representative datasets that accurately mimic realistic scenarios of data gaps. Specifically, we designed four distinct testing scenarios to comprehensively evaluate the robustness of our imputation methods. The first scenario introduces short-term intermittent gaps, randomly masking approximately 5% of MFR sensor measurements. These intermittent gaps typically represent brief operational disruptions, such as transient communication losses or temporary environmental interferences lasting from approximately 30 min up to 1 or 2 h.

The other three scenarios simulate more severe and realistic extended sensor outages, representing continuous periods of missing data lasting 3 days (72 h), 5 days (120 h), and 10 days (240 h), corresponding to 144, 240, and 480 consecutive missing timesteps, respectively. For each of these scenarios, unique continuous intervals were identified for each MFR node across the entire study period. Instead of restricting the analysis to a specific timeframe, we utilized all available data segments that met the quality control criteria. Consequently, while the dataset naturally includes a higher density of samples from the post-March 2025 period due to improved sensor availability, this approach ensures that the model is trained and evaluated on the maximum amount of valid observational data available. All sensor readings within those identified intervals were systematically masked. These prolonged data gaps closely reflect scenarios commonly encountered in operational urban sensor networks, such as extended equipment failures, significant communication disruptions, severe environmental hazards (e.g., flooding, prolonged blockage of transmission signals), or sensor downtime resulting from maintenance and logistical challenges. Consequently, these extended outage scenarios provide a stringent assessment of the imputation methods, testing their effectiveness and resilience under challenging and practically relevant urban environmental monitoring conditions.

## Method of analysis

### Gated attention bi-directional long short-term memory (GA-BiLSTM)

To robustly address the challenge of missing data in urban environmental sensor networks, we propose a modeling framework: the gated attention bidirectional long short-term memory (GA-BiLSTM). This framework is a variant of long short-term memory architecture (Gers et al., 2000; Greff et al., 2016; Yu et al., 2019), strategically integrating advanced temporal modeling capabilities of bidirectional LSTM (Hameed & Garcia-Zapirain, 2020; Lu et al., 2021; Siami-Namini et al., 2019) units with a dynamic, adaptive attention-based gating mechanism (Vaswani et al., 2017; Xue et al., 2020) designed to leverage spatial information from surrounding sensor nodes effectively.

LSTM, a specialized type of recurrent neural network (RNN), is particularly effective for modeling complex sequential relationships in time-series data. Unlike traditional RNNs, which often suffer from vanishing or exploding gradient problems, LSTM networks utilize specialized gating mechanisms—namely, input gates, forget gates, and output gates—to selectively retain or discard information at each timestep. This gating strategy allows LSTMs to effectively model long-range temporal dependencies, making them highly suitable for environmental data, which typically exhibits strong autocorrelation and temporal complexity.

The first stage of this framework involves constructing detailed spatiotemporal input features at each timestep. This is achieved by combining recent temporal observations from the target sensor node with simultaneous spatial observations from surrounding sensor nodes. Specifically, the model includes cyclical temporal encodings, such as sine and cosine transformations of the hour-of-day feature, to effectively capture diurnal cycles. Additionally, it integrates the most recent observations from all available sensor nodes across the network to form comprehensive input vectors.

A critical innovation of this model is the implementation of a gated attention mechanism, which dynamically evaluates and weights spatial information provided by neighboring sensor nodes. Unlike traditional attention mechanisms that apply static weighting schemes, the gated attention approach in this study employs a sophisticated dual-gate structure. One gate calculates attention scores reflecting the relative importance of spatial inputs at each timestep, while the other gate dynamically modulates feature activations based on their contextual significance. This adaptive strategy allows the model to selectively emphasize informative data from spatially correlated sensors and suppress less informative or noisy signals. Crucially, this mechanism addresses the spatial heterogeneity of the network, where sensors are spaced tens of kilometers apart. Instead of relying on fixed distance-based weights, the gated attention mechanism learns to assign high importance scores to distant sensors that exhibit strong environmental coupling (e.g., shared synoptic weather patterns) while down-weighting closer sensors that may be decoupled due to local urban microclimates. Such dynamic feature selection is particularly beneficial in complex urban environments, where the relevance of sensor information may rapidly shift due to transient disturbances or localized environmental conditions.

These carefully constructed features are then processed through a stacked dual-layer bidirectional long short-term memory (BiLSTM) architecture, explicitly designed to exploit both forward (past) and backward (future) temporal contexts. Each bidirectional LSTM layer comprises forward and backward LSTM units that independently process the input sequences from opposite temporal directions. The outputs from these two directions are adaptively integrated through a learned weighting mechanism, effectively synthesizing historical and future insights based on their contextual relevance at each timestep. This capability significantly enhances imputation accuracy, especially for prolonged data gaps ranging from a few days to over a week. By leveraging the LSTM’s ability to maintain long-term dependencies, the model effectively bridges extended outages (e.g., 10 days), preventing the degradation of predictive performance that typically occurs with standard recurrent networks over long sequences.

To further enhance the model’s capacity for capturing intricate environmental patterns, two bidirectional LSTM layers are stacked sequentially. This deep hierarchical structure enables the model to capture a broad spectrum of temporal dependencies, ranging from immediate, short-term fluctuations (on the scale of hours) to more subtle, long-term dynamics spanning days. After traversing these stacked LSTM layers, the resultant high-dimensional temporal and spatial representations are refined through fully connected dense layers, which include dropout regularization to mitigate overfitting. These dense layers ultimately produce precise, context-aware predictions for imputing the missing sensor measurements.

**Fig. 2:**
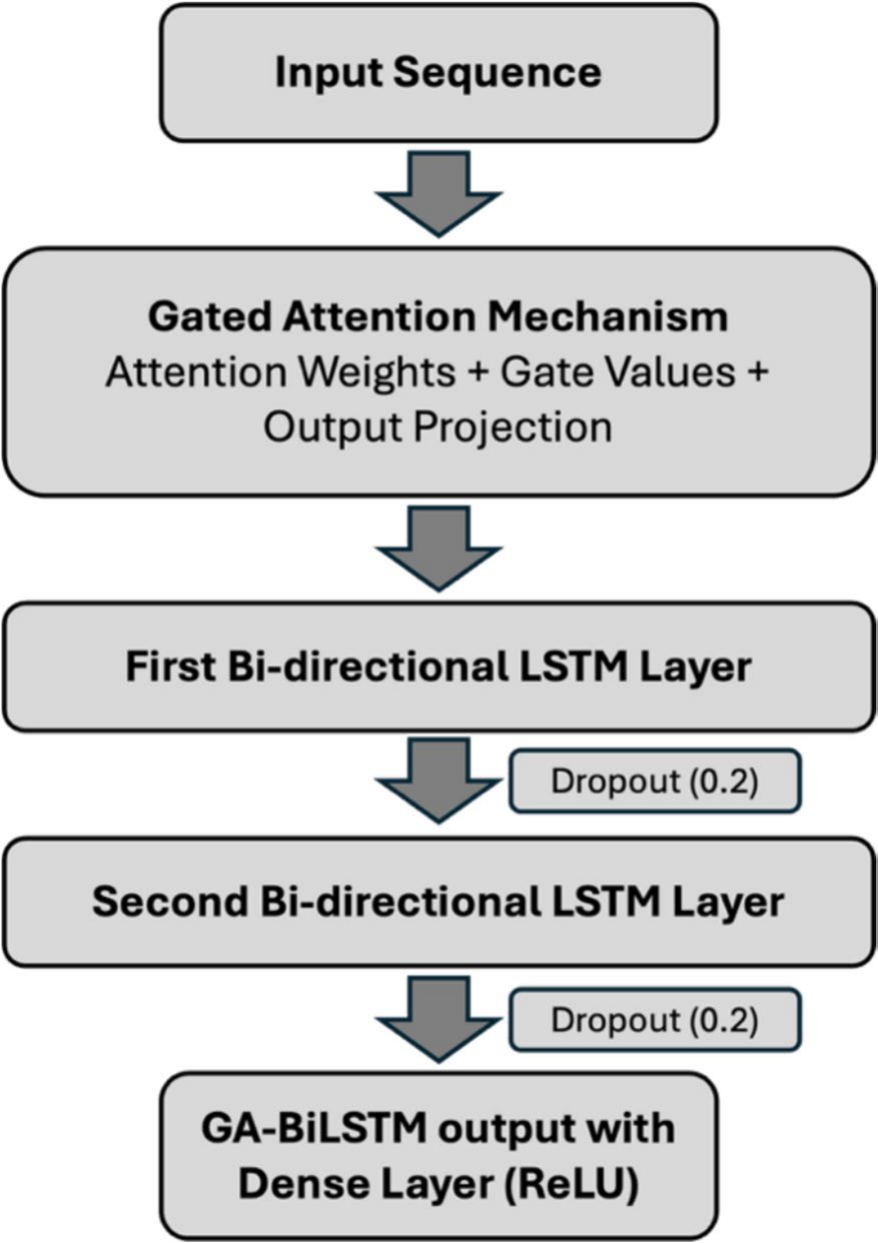
Schematic overview of the gated attention bidirectional LSTM (GA-BiLSTM) model framework for imputing missing environmental sensor data

During the training phase, the model processes spatiotemporal sequences covering 72-h periods (36-h of past and future), integrating both historical and predictive windows. Model parameters are optimized using the Adam optimizer, aiming to minimize the mean squared error (MSE) between observed and predicted sensor values. A validation dataset comprising 20% of the available non-missing data is used to monitor performance. Additionally, a learning-rate scheduler dynamically adjusts the learning rate during training to enhance convergence, and an early stopping strategy based on validation performance ensures robust model generalization. Separate GA-BiLSTM models are trained individually for each target environmental variable and sensor location to ensure optimal performance tailored to distinct temporal and spatial contexts. Figure 2 visually summarizes the comprehensive architecture and data flow of the GA-BiLSTM imputation framework.

To achieve optimal performance, key hyperparameters—including the number of LSTM units, attention size, dropout rate, learning rate, and batch size—were systematically tuned using a grid search approach. The search space included the number of LSTM units [16, 32, 64, 128], attention mechanism dimension [16, 32, 64], dropout rate [0.1, 0.2, 0.3], learning rate [1e-4, 5e-4, 1e-3, 5e-3, 1e-2, 5e-2], batch size [32, 64, 128, 256], and L2 weight decay [1e-5, 1e-4, 1e-3, 1e-2, 0]. The final optimal configuration was identified as follows: 64 LSTM units per layer, an attention dimension of 32, a batch size of 128, a dropout rate of 0.2, and a learning rate of 1e-3 with an L2 regularization factor of 1e-5. Additionally, we utilized the Adam optimizer with a learning rate scheduler that reduced the rate by a factor of 0.5 if validation loss did not improve for 10 epochs (patience).

Crucially, to assess the model’s robustness for real-world deployment where outage durations are unpredictable, we applied this single fixed set of hyperparameters across all testing scenarios (intermittent, 3-day, 5-day, and 10-day outages). This approach ensures that the reported performance reflects the model’s generalized capability rather than being overfitted to specific outage lengths.

### Extreme gradient boosting (XGBoost)

In addition to GA-BiLSTM, our study employs XGBoost (Chen & Guestrin, 2016), an ensemble-based machine learning method. XGBoost constructs predictive models through an ensemble of gradient-boosted decision trees, optimizing predictive accuracy by iteratively fitting new trees to the residual errors of previous ones. It can manage missing values effectively, making it suitable for datasets with frequent or sporadic data gaps, as often encountered in urban environmental sensor networks.

However, XGBoost does not natively handle the temporal nature of sequential data as in LSTM. It treats input features as independent observations, ignoring intrinsic temporal ordering and dependencies crucial in environmental sensor data. To address this limitation and explicitly embed temporal context, our implementation integrates temporal features into the input data. Specifically, we construct additional input variables representing both past and future sensor observations relative to each timestep. These features explicitly capture temporal dependencies, such as recent trends, periodic cycles, and short-term fluctuations, enabling the XGBoost model to approximate temporal relationships indirectly.

For each timestep and each sensor variable, we create a series of lagged features (e.g., measurements 30 min ago, 1 h ago, up to 36 h prior) as well as corresponding lead features (e.g., future measurements 30 min ahead, 1 h ahead, up to 36 h forward). Such bidirectional temporal contextualization significantly enhances the predictive capability of XGBoost by incorporating immediate temporal dynamics surrounding missing data points.

In addition to temporal context, our method incorporates explicit spatial features. These features consist of concurrent measurements from nearby sensor nodes, allowing the model to capture spatial correlations and dependencies inherent in environmental variables. By simultaneously integrating spatial measurements and bidirectional temporal features into the input data, the model effectively leverages the inherent spatiotemporal coherence of urban environmental variables, such as soil moisture and temperature, which typically exhibit similar patterns across adjacent sensor locations.

### K-nearest neighbor (KNN)

As a baseline method for comparison, we employed a K-Nearest Neighbors (KNN) imputation model that relies exclusively on spatial information from the sensor network. Unlike the GA-BiLSTM and XGBoost approaches, KNN does not incorporate temporal context; instead, it estimates missing sensor measurements using concurrent observations from other sensor nodes at the same timestep. Specifically, the imputation process identifies sensors exhibiting similar measurement patterns at each timestep and calculates the missing value based on these spatially proximate observations. To support the identification of relevant neighboring observations, temporal information, such as cyclical hour-of-day encodings, is included, helping the model distinguish basic diurnal patterns.

Prior to imputation, sensor data is standardized to ensure consistent feature scales and facilitate accurate neighbor identification. During training, realistic missing data scenarios are simulated by excluding certain observations, enabling evaluation of the model’s predictive capability. The KNN imputation serves as an intuitive, spatially focused benchmark, highlighting the improvements achievable through more complex methods that explicitly model temporal dependencies.

## Results

### Model comparison

We evaluate and compare the performance of three distinct imputation models (GA-BiLSTM, XGBoost, and KNN) across four different scenarios of missing data: random intermittent gaps lasting approximately 30 min, and prolonged continuous outages spanning 3, 5, and 10 consecutive days. Table 3 summarizes the mean absolute error (MAE) calculated between observed (ground truth) values and model-imputed values. It is important to note that these metrics were computed strictly within the specific “masked” intervals (i.e., the specified 3-day, 5-day, and 10-day periods where data was artificially removed), rather than averaging across the entire deployment timeline. This ensures that the comparison focuses exclusively on the models’ capability to reconstruct missing dynamics during the targeted outage periods, including the rapid diurnal fluctuations characteristic of urban microclimates.

**Table 3:** Mean absolute error (MAE) between observed (ground truth) and imputed values for each environmental variable, averaged across all MFR nodes under the random masking scenario. The best performing model for each outage scenario and variable is marked with bold

Scenario	Variable	GA-BiLSTM	XGBoost	KNN
Random missing	AT (°C)	**3.2082**	4.2367	4.0323
	VPD (kPa)	0.2161	**0.0314**	0.1068
	ST15 (°C)	0.8803	**0.3274**	0.865
	ST30 (°C)	0.5926	**0.2311**	0.678
	ST45 (°C)	0.4614	**0.1808**	0.6654
	ST60 (°C)	0.3589	**0.2083**	0.7265
	VWC15 (m^3^/m^3^)	0.8612	**0.1529**	0.9756
	VWC30 (m^3^/m^3^)	0.605	**0.1199**	0.7824
	VWC45 (m^3^/m^3^)	**0.0902**	0.2515	0.3148
	VWC60 (m^3^/m^3^)	**0.0708**	0.1717	0.406
3-day outage	AT (°C)	**3.6093**	4.6516	5.4509
	VPD (kPa)	0.4856	**0.2236**	0.2474
	ST15 (°C)	**1.7352**	6.1625	3.4025
	ST30 (°C)	**1.1316**	3.7919	2.8197
	ST45 (°C)	**0.9778**	2.3981	2.432
	ST60 (°C)	**0.78**	2.615	2.1576
	VWC15 (m^3^/m^3^)	**1.7021**	3.8936	2.4321
	VWC30 (m^3^/m^3^)	**1.0535**	3.3083	1.4583
	VWC45 (m^3^/m^3^)	**0.3235**	0.9132	0.4265
	VWC60 (m^3^/m^3^)	**0.3645**	1.8173	0.9649
5-day outage	AT (°C)	**3.4048**	4.4945	4.3735
	VPD (kPa)	**0.1285**	0.514	0.1848
	ST15 (°C)	**1.731**	4.399	2.5047
	ST30 (°C)	**1.3966**	2.8871	2.0281
	ST45 (°C)	**1.1623**	1.7575	1.7559
	ST60 (°C)	**1.0505**	1.6488	1.576
	VWC15 (m^3^/m^3^)	**1.4803**	3.3776	2.3551
	VWC30 (m^3^/m^3^)	**1.1321**	1.6774	1.7133
	VWC45 (m^3^/m^3^)	**0.6279**	0.9466	0.7675
	VWC60 (m^3^/m^3^)	**0.3628**	1.0377	0.3805
10-day outage	AT (°C)	**3.4625**	4.0101	4.8429
	VPD (kPa)	0.4221	**0.1885**	0.1962
	ST15 (°C)	**2.3067**	6.9235	2.344
	ST30 (°C)	**2.0283**	4.821	2.0514
	ST45 (°C)	**1.6334**	3.9791	1.9396
	ST60 (°C)	**1.4786**	3.8295	1.8387
	VWC15 (m^3^/m^3^)	**2.9098**	3.8803	3.5521
	VWC30 (m^3^/m^3^)	**2.8822**	3.8656	3.0153
	VWC45 (m^3^/m^3^)	**0.763**	0.9084	1.2246
	VWC60 (m^3^/m^3^)	**0.8462**	1.3467	1.0869

As anticipated, the MAE increases for all models as the duration of data gaps lengthens from short intermittent gaps to extended multi-day outages, reflecting the inherent challenge in accurately reconstructing longer periods of missing sensor data. Under the random intermittent scenario, XGBoost demonstrates slightly better performance compared to GA-BiLSTM. This indicates that for very brief or sporadic data outages, the simpler ensemble-based approach of XGBoost, which relies on immediate local spatial–temporal contexts, provides sufficient predictive accuracy. In these short-gap situations, the bidirectional temporal modeling capabilities of GA-BiLSTM offer relatively limited advantages.

However, when we examine scenarios involving more realistic, prolonged outages (3-day, 5-day, and 10-day gaps), the strengths of GA-BiLSTM become notably apparent. GA-BiLSTM consistently achieves superior performance relative to both XGBoost and KNN across nearly all environmental variables, often by significant margins. This improved performance is due to the ability of GA-BiLSTM to explicitly model complex temporal dependencies from both past and future observations, along with adaptive weighting of spatial correlations across sensor nodes.

### Visual inspection of outage scenarios

**Fig. 3:**
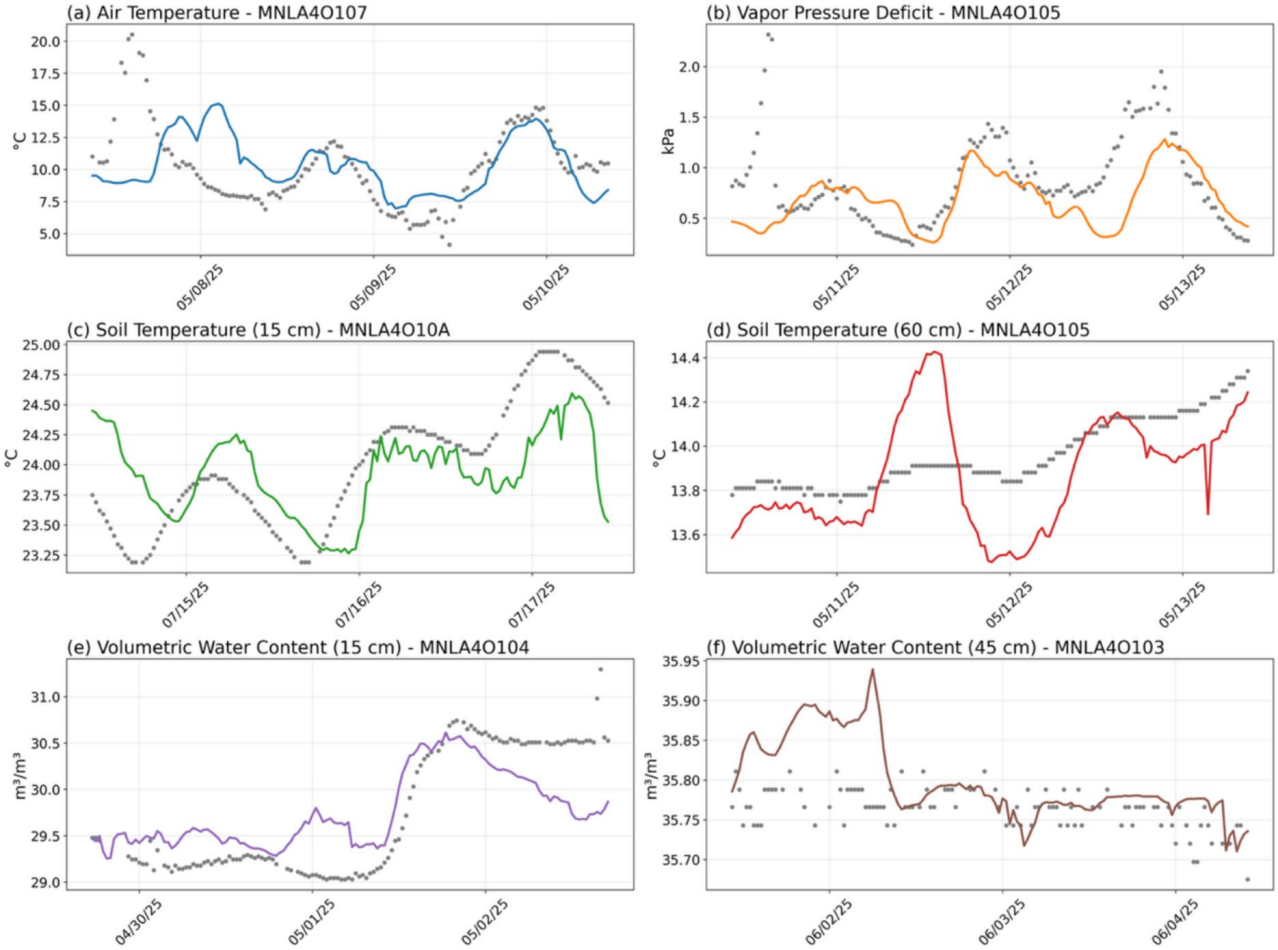
Time series comparisons between observed (ground-truth) sensor values (grey dots) and GA-BiLSTM-imputed values (colored lines) during a continuous 3-day outage period. Selected environmental variables include: **a** air temperature (AT), **b** vapor pressure deficit (VPD), **c** intermediate-depth soil temperature at 30 cm (ST30), **d** deepest soil temperature at 60 cm (ST60), **e** volumetric water content (VWC) at shallow depth of 15 cm (VWC15), and **f** VWC at the deeper depth of 45 cm (VWC45). For each variable, data from the median-performing MFR node (5th best among all nodes) are displayed

**Fig. 4:**
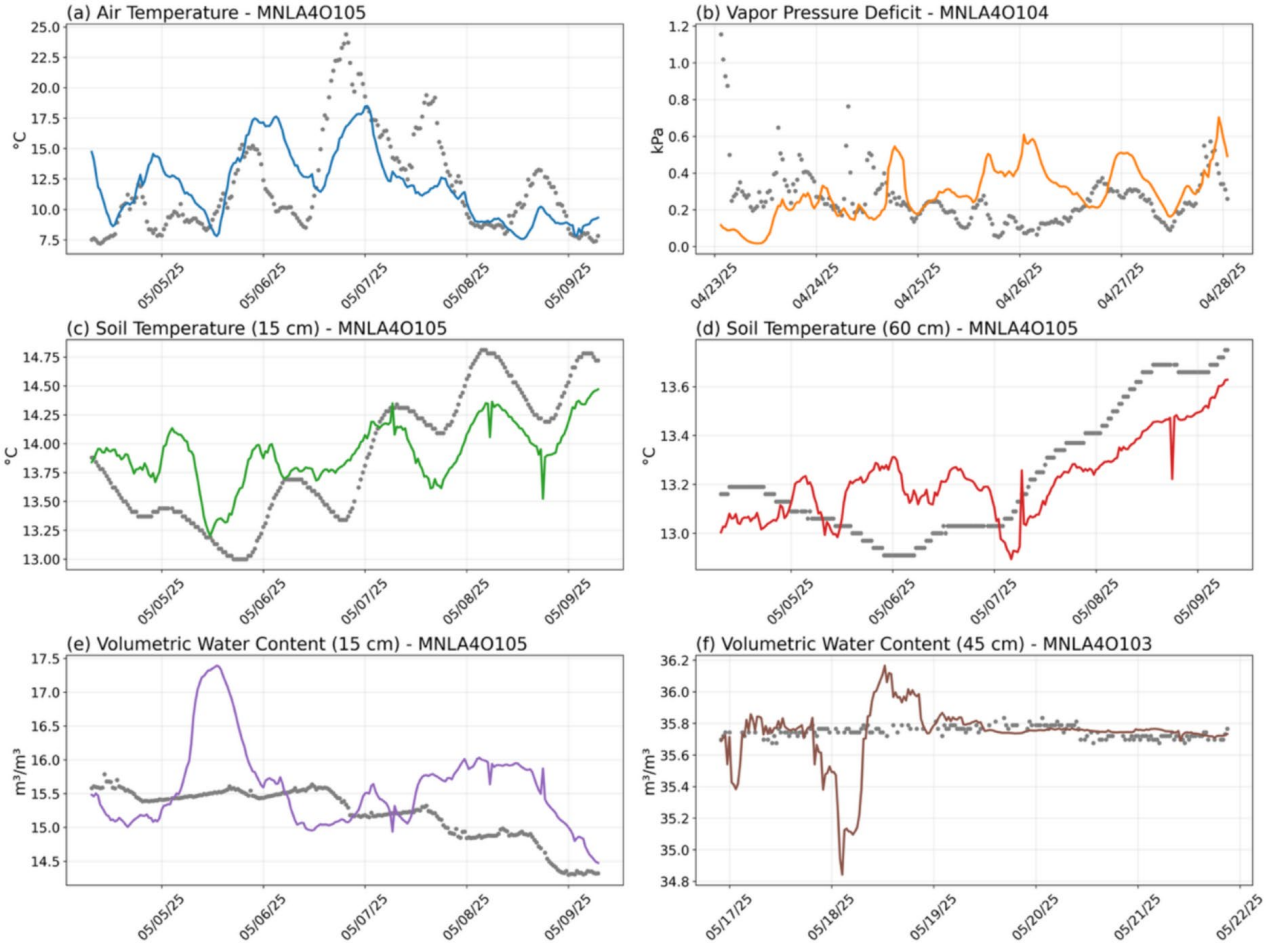
(a–f) Same as Figure 3a–f, but for the 5-day outage scenario

To gain deeper insights into the performance of the GA-BiLSTM model during an extended, realistic outage scenario, we closely examine its imputation accuracy over a continuous 3, 5, and 10-day period. Specifically, we select representative environmental variables—AT, VPD, ST15, ST45, VWC30, VWC60—and visualize imputation results for each at the median-performing MFR node (5th best performing node out of 9). These selected time series, along with their respective ground-truth measurements, are shown in Figs. 3, 4, and to 5.

**Fig. 5:**
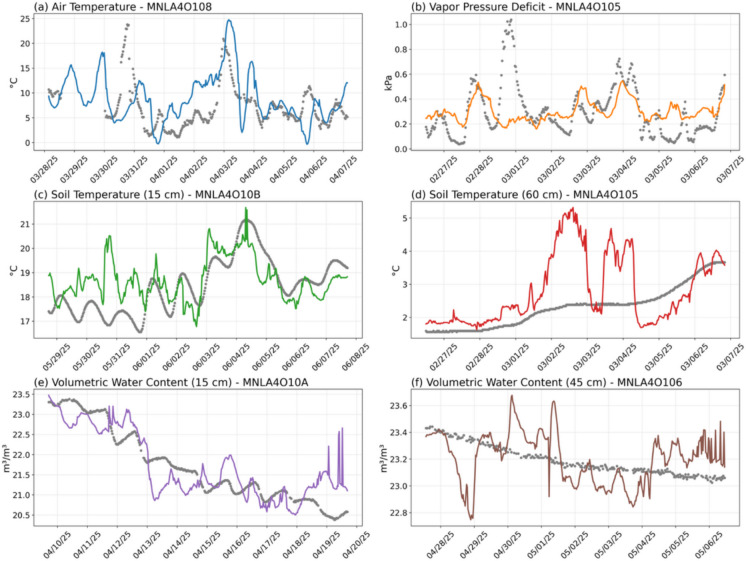
(a–f) Same as Figure 3a–f, but for the 10-day outage scenario

Overall, visual inspection of the GA-BiLSTM imputation results for the 3-day, 5-day, and 10-day outage scenarios highlights several notable insights. The model robustly captures key temporal patterns in environmental variables, such as AT and VPD closely following diurnal fluctuations and accurately replicating both rapid and gradual changes.

For soil temperature measurements at various depths (intermediate at 30 cm and deeper at 60 cm), the GA-BiLSTM exhibits strong overall performance, effectively tracking major trends and fluctuations. Nevertheless, at deeper soil depths, the model occasionally shows reduced accuracy, reflecting inherent difficulties due to muted temporal variability at these layers.

For VWC, while the model successfully identifies general trends and sharp transitional events (such as sudden increases or decreases), it frequently either underestimates or overestimates absolute VWC values. This systematic bias appears particularly prominent during prolonged outages, highlighting the challenge in accurately predicting low-variance soil moisture dynamics without consistent observational context.

Notably, the accuracy of imputation is highest near the temporal boundaries of the outage—specifically within the first and last 24 h of the gap. This enhancement results from the GA-BiLSTM’s inherent bidirectional temporal structure, which leverages the immediate observational context just before the outage begins and just after it ends. Consequently, predictions during these boundary periods consistently outperform those at the temporal center (the middle 24 to 48 h), where the temporal distance from ground-truth observations is maximized. This pattern demonstrates the critical importance of proximity to surrounding observational data for maintaining high predictive fidelity.

### Cross-site feature importance for MFR sensor variables

To gain deeper insights into the spatial dependencies influencing the performance of GA-BiLSTM imputation, we conducted a comprehensive cross-site feature importance analysis utilizing permutation importance. This method quantifies each predictor’s contribution by measuring the decrease in model accuracy when that variable is randomly permuted. Each predictor was permuted 100 times to ensure statistically robust conclusions. Importantly, to isolate purely spatial relationships, we explicitly excluded any features measured at the same site as the target variable, thus evaluating only cross-site (network-wide) relationships. These importance scores directly reflect the effectiveness of the Gated Attention mechanism in identifying relevant spatial predictors across the dispersed network, validating the method’s design against the varying spatial distances between nodes. This means that predictors could represent the same type of environmental measurement as the target variable (e.g., soil temperature at different depths) but were always sourced from other sensor locations throughout the network. Table 4 summarizes the three most influential cross-site predictors for each MFR sensor variable.

**Table 4:** Top three most important cross-site predictor variables identified by permutation feature importance for each target MFR node variable. Importance scores were averaged across multiple MFR nodes, explicitly excluding predictor variables from the same site to highlight purely spatial (cross-site) relationships. Variable names indicate measurement type followed by sensor type in parentheses

Target variable	First feature	Second feature	Third feature
AT	Wind speed (WXT)	Wind direction (WXT)	Relative humidity (BME280)
VPD	Wind direction (WXT)	Ozone (AQT)	NO (AQT)
ST15	Relative humidity (BME280)	Wind direction (WXT)	Wind speed (WXT)
ST30	Relative humidity (BME280)	Wind speed (WXT)	Wind direction (WXT)
ST45	Relative humidity (BME280)	Wind speed (WXT)	Wind direction (WXT)
ST60	Relative humidity (BME280)	Wind speed (WXT)	Wind direction (WXT)
VWC15	Relative humidity (BME280)	Wind speed (WXT)	Wind direction (WXT)
VWC30	Wind speed (WXT)	NO (AQT)	Wind direction (WXT)
VWC45	Ozone (AQT)	Wind speed (WXT)	Relative humidity (BME280)
VWC60	Wind speed (WXT)	Relative humidity (BME280)	VWC (15 cm) (MFR)

AT imputation across all MFR nodes was primarily influenced by wind speed, wind direction, and relative humidity measured by other sensors across the network. These results underline the strong coupling between airflow patterns and atmospheric moisture conditions across the monitoring network, reflecting the spatial coherence of urban microclimatic conditions at a regional scale.

For VPD spatial predictors related to wind direction, ozone, and nitric oxide (NO) concentrations showed substantial importance. Particularly noteworthy is the prominent role of air quality variables (ozone and NO) as predictive features. However, it is critical to emphasize that these relationships indicate correlations rather than direct causation. The association between VPD and air pollutants likely arises because both are influenced simultaneously by overarching synoptic meteorological conditions rather than any direct causal influence.

ST at multiple depths (15 cm, 30 cm, 45 cm, and 60 cm) consistently showed high predictive dependency on relative humidity, wind speed, and wind direction from across the monitoring network. This spatial correlation likely reflects the integrated response of urban soils to broader regional weather conditions, particularly moisture and heat transport facilitated by wind.

Imputation of VWC exhibited more varied spatial predictors. Relative humidity, wind speed, and air pollutants (ozone and NO) consistently appeared as important predictive variables. Additionally, VWC at 60 cm depth uniquely showed dependency on shallower VWC (15 cm), suggesting vertically coherent moisture dynamics within the soil profile. Nonetheless, similar to air quality variables, these cross-site relationships involving air pollutants must again be interpreted strictly as correlations, likely reflecting shared meteorological conditions driving both soil moisture variations and air quality patterns rather than direct causal interactions.

### Network analysis of spatial information flow

Leveraging the results from the permutation importance analysis, we conduct network analysis to examine spatial patterns of information flow between Waggle and MFR nodes distributed across the Chicago metropolitan area. The goal of the network analysis is to quantify the directional strength of information flow for each node.

**Fig. 6:**
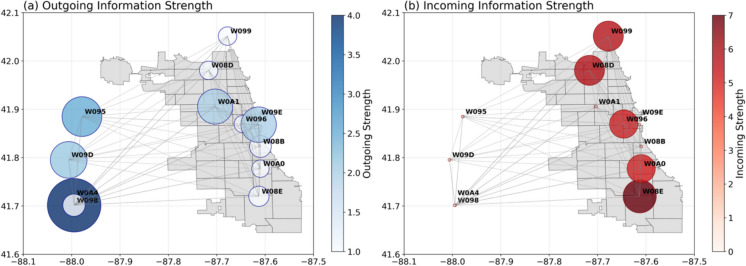
Spatial visualization of network strength and spatial interdependencies among urban environmental sensor nodes in the Chicago metropolitan area. Each node is depicted as a circle geographically positioned according to its actual coordinates, with circle size and color representing the (**a**) outgoing information strength and (**b**) incoming information strength for imputation of MFR nodes

For each node, we compute outgoing information strength by summing permutation importance scores that represent how crucial its data is for accurately imputing environmental variables at all other sensor locations. Conversely, incoming information strength sums permutation importance scores representing how strongly each node depends on predictive information from other nodes. To ensure a fair and unbiased comparison among nodes despite varying lengths of their operational periods, we normalize these summed importance scores by each node’s total duration of available data. This normalization is done to highlight sensor locations with intrinsically greater predictive significance within the network, independent of their deployment timing. The results are presented in Fig. 6.

Analyzing outgoing information strengths reveals that rural nodes (W098, W095, W09D) contribute the highest predictive value across the sensor network. To quantitatively assess the importance of these rural nodes, we conducted an additional sensitivity analysis in which each node was individually disabled, and we measured the resulting deterioration in imputation accuracy (Table 5), considering only periods following the installation of these nodes. Disabling any one of these three rural nodes significantly increases the MAE of the GA-BiLSTM imputation model compared to the complete network scenario, averaged across all environmental variables and outage scenarios. Specifically, disabling a single rural node increases the MAE by approximately 10%, while disabling all three simultaneously results in an MAE increase exceeding 50%.

**Table 5:** Increase in mean absolute error (MAE) of the GA-BiLSTM imputation model when individually disabling rural Waggle nodes W098, W095, and W09D. Values represent the average relative increase in MAE across all environmental variables and outage scenarios

MFR variable	W098	W095	W09D
AT (°C)	0.2373	0.2052	0.1544
VPD (kPa)	0.2425	0.2393	0.2216
ST15 (°C)	0.2504	0.1433	0.1021
ST30 (°C)	0.1332	0.1161	0.0629
ST45 (°C)	0.0659	0.0419	0.0402
ST60 (°C)	0.0222	0.0013	0.0063
VWC15 (m^3^/m^3^)	0.0468	0.0704	0.0548
VWC30 (m^3^/m^3^)	0.0805	0.0419	0.0214
VWC45 (m^3^/m^3^)	0.0319	0.0173	0.0067
VWC60 (m^3^/m^3^)	0.0384	0.0253	0.0245

One might anticipate that urban nodes, due to their geographic proximity to target sensor locations, would provide the strongest predictive relationships. However, our results demonstrate the opposite, clearly showing that rural nodes consistently exhibit greater predictive influence. This is because urban meteorological conditions are influenced by numerous localized factors such as building structures, vehicular traffic dynamics, and urban vegetation, creating substantial variability even at fine spatial scales. Thus, geographic proximity among urban sensor nodes does not necessarily translate into strong predictive relationships or information exchanges. Conversely, rural sensor nodes provide environmental measurements less impacted by complex urban phenomena, resulting in more stable and reliable data streams. These rural measurements effectively capture broader-scale meteorological and synoptic conditions, making them particularly valuable predictors for urban conditions, especially in scenarios involving extensive missing data. Consequently, strategically expanding the sensor network to enhance rural or peri-urban node density could markedly improve data imputation accuracy and overall resilience in urban environmental monitoring systems.

Examining incoming information strengths reveals that all MFR nodes exhibit comparable dependencies on network information, emphasizing their roles as specialized hubs that aggregate information from across the entire sensor network to reconstruct local environmental conditions accurately.

### Operational uncertainty estimation

To enhance the operational value of the GA-BiLSTM model, it is crucial to provide not just point estimates but also a measure of reliability. Since the proposed model is deterministic, we estimated operational uncertainty by analyzing the distribution of prediction residuals derived from the validation dataset. Assuming the errors follow a normal distribution, we calculated the standard deviation (σ) of the residuals to construct an empirical 95% confidence interval (1.96σ) for the imputed values (Table 6). These uncertainty metrics allow urban environmental managers to assess the trustworthiness of the filled data in real-time. For instance, imputed values falling within physically critical ranges can be flagged for manual review if their uncertainty bounds exceed acceptable safety thresholds. This empirical approach provides a computationally efficient method for uncertainty quantification suitable for real-time deployment.

**Table 6:** 95% confidence interval (1.96 standard deviation) of the validation dataset for each variable, for each scenario

Scenario	Variable	GA-BiLSTM based Confidence interval (1.96σ)
Random missing	AT (°C)	5.0690
	VPD (kPa)	0.4733
	ST15 (°C)	2.1127
	ST30 (°C)	0.9126
	ST45 (°C)	1.1212
	ST60 (°C)	0.5958
	VWC15 (m^3^/m^3^)	1.4038
	VWC30 (m^3^/m^3^)	1.2947
	VWC45 (m^3^/m^3^)	0.2246
	VWC60 (m^3^/m^3^)	0.1423
3-day outage	AT (°C)	6.3524
	VPD (kPa)	0.7333
	ST15 (°C)	2.8978
	ST30 (°C)	2.6479
	ST45 (°C)	1.6525
	ST60 (°C)	1.9344
	VWC15 (m^3^/m^3^)	3.1829
	VWC30 (m^3^/m^3^)	1.7277
	VWC45 (m^3^/m^3^)	0.5176
	VWC60 (m^3^/m^3^)	0.5978
5-day outage	AT (°C)	7.1160
	VPD (kPa)	0.2107
	ST15 (°C)	4.0159
	ST30 (°C)	2.7653
	ST45 (°C)	2.2897
	ST60 (°C)	1.7228
	VWC15 (m^3^/m^3^)	2.9162
	VWC30 (m^3^/m^3^)	2.1623
	VWC45 (m^3^/m^3^)	1.1177
	VWC60 (m^3^/m^3^)	0.5623
10-day outage	AT (°C)	7.6175
	VPD (kPa)	1.0468
	ST15 (°C)	5.3054
	ST30 (°C)	4.8882
	ST45 (°C)	3.0545
	ST60 (°C)	2.9276
	VWC15 (m^3^/m^3^)	6.4016
	VWC30 (m^3^/m^3^)	6.2256
	VWC45 (m^3^/m^3^)	1.4116
	VWC60 (m^3^/m^3^)	1.9293

### Mechanistic roles of model components

The performance of the GA-BiLSTM can be attributed to the distinct yet complementary roles of its two core components: the Gated Attention mechanism and the Bidirectional LSTM structure. Our analysis of the results confirms how these components work independently to address specific imputation challenges.

First, the Gated Attention mechanism functions as a spatial filter. As evidenced by the cross-site feature importance analysis (the “Cross-site feature importance for MFR sensor variables” section), this component allows the model to dynamically identify and prioritize spatially informative nodes—such as the distant rural sensors (W098, W095)—regardless of their physical proximity. Without this attention-based spatial weighting, the model would treat all neighbors equally or rely solely on distance, failing to capture the network-wide synoptic patterns crucial for urban imputation.

Second, the Bidirectional logic serves as a temporal bridge. The visual inspection of extended outages (the “Visual inspection of outage scenarios” section) revealed that prediction accuracy is significantly higher near the outage boundaries compared to the center. This behavior demonstrates the independent contribution of the bidirectional architecture, which propagates valid information from both the past (forward pass) and the future (backward pass) into the missing interval. This capability is distinct from the spatial attention and is essential for maintaining continuity during prolonged data gaps where spatial correlations alone may be insufficient.

## Summary and conclusions

In this study, we developed a gated attention bidirectional long short-term memory (GA-BiLSTM) model to effectively address the issue of missing data within urban environmental sensor networks. By leveraging spatial correlations between sensor nodes and bidirectional temporal dependencies in sensor data, GA-BiLSTM demonstrated significantly superior imputation performance compared to conventional methods (XGBoost and KNN), particularly during extended sensor outages lasting up to 10 consecutive days. Evaluations conducted on data from the CROCUS urban sensor network in Chicago underscored the capability of GA-BiLSTM to reconstruct complex surface and subsurface environmental conditions, critical to urban monitoring and management.

Our results offer several significant insights. First, the marked improvement in model performance observed during prolonged outage scenarios emphasizes the value of explicitly modeling bidirectional temporal dynamics. Unlike unidirectional or non-sequential models, GA-BiLSTM captures critical contextual information from both preceding and subsequent observations surrounding missing intervals, resulting in notably improved accuracy, especially near the boundaries of data gaps. This capability is particularly valuable given the dynamic and heterogeneous nature of urban environments, characterized by complex spatial–temporal interactions and rapid environmental fluctuations.

Second, our feature importance and spatial network analyses revealed meaningful predictive roles for rural or peripheral sensor nodes. Specifically, measurements from rural locations, minimally affected by urban disturbances such as heat islands, anthropogenic activities, and built-environment effects, proved exceptionally effective in filling data gaps for sensors located within urban areas. This key finding indicates that geographic proximity alone does not dictate the strength of sensor correlations, highlighting instead the strategic benefit of integrating stable, high-quality rural sensor data into urban environmental monitoring frameworks. Our analyses identified nodes such as W098, W095, and W09D as especially influential for accurately imputing environmental conditions in the urban interior. Despite their predictive importance, rural nodes often face lower operational priority due to logistical challenges such as greater travel distances, maintenance complexity, and resource constraints at the managing institutions. Nevertheless, our analysis demonstrates that preserving these rural nodes—and thus the complete integrity of the sensor network—is essential to maintaining the overall robustness, accuracy, and continuity of urban environmental monitoring.

Third, beyond the individual importance of sensor nodes, our findings emphasize the necessity of strategically placing sensors in configurations that optimize complementary predictive capabilities. Network performance does not rely solely on the reliability of individual sensors but also on their synergistic arrangement, which collectively enhance predictive accuracy and reduces vulnerability to data outages. The analytical framework presented in this study provides quantitative insights that can guide decision-makers in the optimal placement of new sensors, the prioritization and maintenance of existing nodes, and in ensuring sufficient sensor density. This strategic deployment ensures both individual node robustness and network-wide resilience, significantly improving the continuity and effectiveness of environmental monitoring.

Future research directions can include broadening the range of sensor technologies integrated within monitoring networks—for instance, incorporating eddy covariance flux towers, advanced air-quality sensors, or fiber optic sensing techniques. Such diversified sensor integration would enhance predictive capabilities, reduce model uncertainties, and deliver richer, multi-dimensional contextual information essential for capturing the intricacies of urban environments. Additionally, systematic investigations into optimal sensor density, spatial distribution, and innovative combinations of stationary and mobile monitoring platforms, such as drone-based or vehicle-mounted sensors, would be highly beneficial for developing more robust, flexible, and adaptive urban monitoring strategies.

Moreover, exploring the generalizability and scalability of the GA-BiLSTM model across various urban contexts is critical to understanding its performance boundaries. Conceptually, the model’s effectiveness relies on the existence of spatiotemporal coherence within the sensor network. In cities with dense high-rise morphologies (e.g., New York City or Hong Kong), where deep street canyons create highly fragmented microclimates, the spatial correlation length may drop below the average inter-sensor distance. Under such structural conditions, the Gated Attention mechanism may struggle to identify informative neighbors, forcing the model to rely predominantly on temporal interpolation, which could reduce accuracy during extended outages. Similarly, regarding environmental conditions, while the model is expected to perform robustly in climates with stable diurnal cycles (e.g., arid regions), its performance may encounter boundaries in tropical environments characterized by highly stochastic, localized convective storms that disrupt temporal continuity. Future studies should therefore investigate transfer learning and domain adaptation approaches, facilitating rapid and efficient deployment of the GA-BiLSTM methodology across diverse urban environments and thus maximizing its operational versatility and reliability.

Another important future research area involves rigorously assessing the practical implementation of GA-BiLSTM models within real-time monitoring systems. Given the computational demands intrinsic to deep learning methods, future research should focus on evaluating and optimizing computational efficiency. Investigating cloud-based or edge-computing architectures will help enable real-time environmental monitoring, facilitate rapid decision-making, and support timely responses in hazard mitigation, emergency management, and adaptive urban environmental planning.

In conclusion, this study significantly advances methodological approaches for effectively managing missing data challenges in urban environmental monitoring, highlighting the critical importance of explicitly modeling spatial and temporal relationships. The insights presented offer concrete, data-driven guidance for optimizing sensor deployment, strategic maintenance, and network resilience, thereby laying a robust foundation for enhanced urban environmental sustainability and adaptability. Continued research addressing sensor deployment strategies, uncertainty quantification, model transferability, and real-time operationalization will further enhance these contributions, ultimately supporting the development of more resilient, adaptive, and sustainable urban ecosystems.

## Data Availability

CROCUS sensor measurement used in this study are available upon request.
